# Endurance exercise training programs intestinal lipid metabolism in a rat model of obesity and type 2 diabetes

**DOI:** 10.14814/phy2.12232

**Published:** 2015-01-19

**Authors:** Yu‐Han Hung, Melissa A. Linden, Alicia Gordon, R. Scott Rector, Kimberly K. Buhman

**Affiliations:** Department of Nutrition Science, Purdue University, West Lafayette, Indiana; Department of Nutrition and Exercise Physiology, University of Missouri, Columbia, Missouri; Research Service, Harry S Truman Memorial Veterans Hospital, Columbia, Missouri; Department of Clinical Medicine, University of Dublin, Dublin, Ireland; School of Biological Sciences, Dublin Institute of Technology, Dublin, Ireland; Division of Gastroenterology and Hepatology, Department of Medicine, University of Missouri, Columbia, Missouri

**Keywords:** Endurance exercise, lipid metabolism, obesity, Otsuka Long‐Evans Tokushima Fatty rat, small intestine

## Abstract

Endurance exercise has been shown to improve metabolic outcomes in obesity and type 2 diabetes; however, the physiological and molecular mechanisms for these benefits are not completely understood. Although endurance exercise has been shown to decrease lipogenesis, promote fatty acid oxidation (FAO), and increase mitochondrial biosynthesis in adipose tissue, muscle, and liver, its effects on intestinal lipid metabolism remain unknown. The absorptive cells of the small intestine, enterocytes, mediate the highly efficient absorption and processing of nutrients, including dietary fat for delivery throughout the body. We investigated how endurance exercise altered intestinal lipid metabolism in obesity and type 2 diabetes using Otsuka Long‐Evans Tokushima Fatty (OLETF) rats. We assessed mRNA levels of genes associated with intestinal lipid metabolism in nonhyperphagic, sedentary Long‐Evans Tokushima Otsuka (LETO) rats (L‐Sed), hyperphagic, sedentary OLETF rats (O‐Sed), and endurance exercised OLETF rats (O‐EndEx). O‐Sed rats developed hyperphagia‐induced obesity (HIO) and type 2 diabetes compared with L‐Sed rats. O‐EndEx rats gained significantly less weight and fat pad mass, and had improved serum metabolic parameters without change in food consumption compared to O‐Sed rats. Endurance exercise resulted in dramatic up‐regulation of a number of genes in intestinal lipid metabolism and mitochondrial content compared with sedentary rats. Overall, this study provides evidence that endurance exercise programs intestinal lipid metabolism, likely contributing to its role in improving metabolic outcomes in obesity and type 2 diabetes.

## Abstract (precis)

Endurance exercise has been shown to improve metabolic outcomes in obesity and type 2 diabetes; however, the physiological and molecular mechanisms for these benefits are not completely understood. Although endurance exercise has been shown to decrease lipogenesis, promote fatty acid oxidation (FAO), and increase mitochondrial biosynthesis in adipose tissue, muscle, and liver, its effects on intestinal lipid metabolism remain unknown. Endurance exercise resulted in dramatic up‐regulation of a number of genes in intestinal lipid metabolism and mitochondrial content compared with sedentary rats. Overall, this study provides evidence that endurance exercise programs intestinal lipid metabolism, likely contributing to its role in improving metabolic outcomes in obesity and type 2 diabetes.

## Introduction

The obesity epidemic is associated with the overconsumption of energy‐dense dietary fat, higher calorie intake, and a sedentary lifestyle in developed countries (Hedley et al. 2004; Cordain et al. 2005; Matthews et al. 2008). In addition, obesity increases the risk of developing other metabolic diseases including type 2 diabetes mellitus, hepatic steatosis, and cardiovascular diseases (Friedman 2002; Nikolopoulou and Kadoglou 2012). Endurance exercise (EndEx) has been shown to improve metabolic outcomes in obesity and type 2 diabetes in human studies; however, the physiological and molecular mechanisms for these benefits are not completely understood. The beneficial effects of EndEx include weight loss (Cuff et al. 2003), improved insulin sensitivity and glucose metabolism (O'Hagan et al. 2013), ameliorated postprandial triglyceridemic response (Merrill et al. 1989), and increased rate of fat oxidation (Bordenave et al. 2008). However, it remains unclear how EndEx training may affect lipid metabolism in the small intestine.

The small intestine mediates highly efficient absorption and processing of dietary fat for delivery throughout the body (Mansbach and Gorelick 2007). Once inside the enterocytes, the absorptive cells of the small intestine, digested products of dietary fat are resynthesized into triacylglycerol (TAG), which are packaged in chylomicrons (CMs) for secretion into lymph and then circulation or are packaged in cytoplasmic lipid droplets (CLDs) (Zhu et al. 2009; Uchida et al. 2012). The TAG that is, stored in CLDs is cleared from the enterocytes after fasting, likely the result of TAG secretion and/or fatty acid oxidation (FAO) within enterocytes. After a meal, TAG levels in blood increase due to an increase in TAG secretion from the small intestine and then decrease over time as a result of clearance of TAG by peripheral tissues. This balance contributes to postprandial TAG levels and energy distribution throughout the body.

The Otsuka Long‐Evans Tokushima Fatty (OLETF) rat is a commonly studied model of obesity and type 2 diabetes (Kawano et al. 1992) that has been used to investigate the therapeutic effects of EndEx on obesity and type 2 diabetes. Selectively bred for deficiency in cholecystokinin‐1 receptor, OLETF rats lack within‐meal feedback for mediating satiety and exhibit hyperphagia‐induced progressive development of obesity, insulin resistance, and type 2 diabetes (Moran et al. 1998; Rector et al. 2010a). The therapeutic effects of EndEx on programming lipid metabolism have been examined in many tissues in OLETF rats. In contrast to hyperphagia‐induced obesity (HIO), which increases fat deposition in both adipose and ectopically in nonadipose tissues, EndEx suppresses lipogenesis and promotes mitochondrial FAO in the liver (Rector et al. 2008) and adipose tissue (Laye et al. 2009). However, lipogenesis in skeletal muscle remains the same with EndEx (Rector et al. 2010b). In addition, EndEx increases mitochondria content in the liver (Rector et al. 2011), adipose tissue (Laye et al. 2009), and skeletal muscle (Rector et al. 2010b), allowing more efficient FAO in response to the large energy demand during exercise. A previous study in OLETF rats demonstrated that HIO increased and EndEx decreased TAG secretion from the small intestine (Hayashi et al. 2002); however, how HIO and EndEx program intestinal lipid metabolism remains unclear.

Given that EndEx programs lipid metabolism in adipose, muscle, and liver and that the small intestine mediates processing dietary fat for systemic delivery and modifies the risk of development of obesity and diabetes, we hypothesized that EndEx programs intestinal lipid metabolism that is dysregulated in HIO to reduce obesity and type 2 diabetes. To determine the effects of EndEx on intestinal lipid metabolism, we assessed the mRNA levels of genes associated with intestinal lipid metabolism in control, nonhyperphagic, sedentary Long‐Evans Tokushima Otsuka (LETO) rats (L‐Sed), hyperphagic, sedentary OLETF rats (O‐Sed), and EndEx trained OLETF rats (O‐EndEx). Although levels of the proteins and their function in intestinal lipid metabolism are ultimately essential for confirming the effects of exercise on intestinal lipid metabolism, mRNA levels have been previously associated with functional changes in intestinal lipid metabolism (van Schothorst et al. 2009; Uchida et al. 2011; Kimura et al. 2013).

## Materials and Methods

### Animals and experimental design

Male LETO and OLETF rats were obtained at 4 weeks of age (Tokushima Research Institute, Otsuka Pharmaceutical, Tokushima, Japan). Animals were individually housed in a temperature‐controlled (21°C) environment with 0600–1800 h light and 1800–0600 h dark cycles. All groups had ad libitum access to water and standard chow which was composed of 56% carbohydrate, 17% fat, and 27% protein (Formulab 5008; Purina Mills, St Louis, MO). The rats were assigned to three groups (*n* = 6–8 rats in each group): 1) sedentary LETO (L‐Sed), 2) sedentary OLETF (O‐Sed) and 3) EndEx OLETF (O‐EndEx). L‐Sed rats were used as healthy, nonhyperphagic controls and O‐Sed rats were hyperphagic and developed obesity and type 2 diabetes. At 19 weeks of age, all OLETF rats were exposed to treadmill running at 15 m/min for 5 min/day to allow for acclimation to the running stimulus. OLETF rats were then randomly assigned to O‐Sed or O‐EndEx. Treatments began at 20 weeks of age, because it has previously documented that the OLETF rat becomes hyperglycemic and hyperinsulinemic at this age (Bunker et al. 2010; Rector et al. 2010a). EndEx training initially consisted of treadmill running at a speed of 15 m/min on a 15% incline for 5 min/day. Duration and speed were gradually increased by 2–3 min/day and 1–2 m/min per week such that by week 4 the animals were running at a speed of 20 m/min on a 15% incline for 60 min/day, 5 days/week and this intensity was maintained for the duration of the treatment. Exercise training lasted 12 weeks and all the animals were killed at age 32 weeks. The last exercise bout was performed 18 h prior to euthanasia. Food was removed from the cages 12 h prior to death, and water was removed on the morning of the experiment 1 h prior to euthanasia. All protocols were approved by the University of Missouri Animal Care and Use Committee. The animals were killed by exsanguination and intestinal mucosa and blood were then collected. The small intestine was divided into six equal length segments and labeled S1–S6 (proximal to distal) in relation to the stomach. S1 represented the duodenum and S2 represents upper jejunum (Uchida et al. 2013).

### Body weight, body composition, and food intake

Body weights and food intakes were monitored and recorded on a weekly basis. Body composition was assessed by dual‐energy X‐ray absorptiometry (DXA; Hologic QDR‐1000, Bedford, MA; calibrated for rodents) on the day of death. Omental, retroperitoneal, and epididymal fat pads were collected and weighed.

### Blood parameters

Whole blood was collected on the day of euthanasia and serum samples were prepared by centrifugation and stored at −80°C until analysis. Glucose and TAG assays were performed by a commercial laboratory (Comparative Clinical Pathology Services, Columbia, MO) on an Olympus AU680 automated chemistry analyzer (Beckman‐Coulter, Brea, CA) using commercially available assays according to manufacturers’ guidelines. Plasma insulin concentrations were determined using a commercially available, rat‐specific enzyme‐linked immunosorbent assay (Alpco Diagnostics, Salem, NH). Samples were run in duplicate and manufacturers’ controls and calibrators were used according to assay instructions.

### RNA extraction and cDNA synthesis

Total RNA was extracted from S2 of intestinal mucosa using RNA Stat‐60 (Tel‐Test, Friendswood, TX). The RNA was quantified using NanoDrop ND‐1000 (Thermo Fischer Scientific, Wilmington, DE) then DNase treated with TurboDNA‐free (Ambion, Austin, TX). cDNA was synthesized from 1 *μ*g Dnase treated RNA by Affinity Script QPCR cDNA using oligo dT and random hexamer primers (Stratagene, LaJolla, CA).

### q‐PCR measurement

SYBR green q‐PCR was performed using Mx3000P QPCR System (Stratagene) and Brilliant II and III SYBR green mastermix (Stratagene). Primers were produced by Integrated DNA technologies (Coralville, IA) and designed to generate PCR products between 100 and 400 base pairs. Primers used for this study are as shown in Table 1. The genes that were examined include acetyl‐CoA carboxylase 1 (*Acc1)*, acyl‐CoA synthetase 5 (*Acsl5*), acyl‐CoA oxidase (*Acox*), adipose triglyceride lipase (*Atgl*), fatty acid translocase (*Cd36*), carnitine palmitoyltransferase I (*Cpt1*), diacylglycerol‐O‐acyltransferase 1 (*Dgat1*), diacylglycerol‐O‐acyltransferase 2 (*Dgat2*), hormone‐sensitive lipase (*Hsl*), liver X receptor *α* (*Lxrα*), medium‐chain acyl‐coenzyme A dehydrogenase (*Mcad*), monoacylglycerol‐O‐acyltransferase 2 (*Mgat2*), microsomal triglyceride transfer protein (*Mtp*), perilipin 2 (*Plin2*), perilipin 3 (*Plin3*), peroxisome proliferator‐activated receptor *α* (*Pparα*), PPAR‐*γ* coactivator‐1 *α* (*Pgc1α*), sterol regulatory element‐binding protein 1 (*Srebp1c*), mitochondria transcription factor A (*Tfam*), and uncoupling protein 2 (*Ucp2*). Each primer was validated for producing a single product of the correct size and amplifying product with efficiency between 80 and 120%. Analysis of the melting curve confirmed the production of a single product and post‐PCR products were subjected to 1.5% agarose gel electrophoresis for ensuring a single product and that the product size was correct. The mRNA level of each gene was normalized to18S mRNA levels and calculated with the comparative CT method using L‐Sed as the reference group. In addition, there is no significant difference in CT values of 18S among experimental groups.

**Table 1.** Primers used for q‐PCR.

Gene	Forward	Reverse
*18S*	5′‐TTAGAGTGTTCAAAGCAGGCCCGA‐3′	5′‐TCTTGGCAAATGCTTTCGCTCTGG‐3′
*Acsl5*	5′‐CTGACACCGACACTGAAAG‐3′	5′‐GACTGAAGTCCAAGGAGAAAG‐3′
*Acc1*	5′‐CGGTCACACTTCACTCTATG‐3′	5′‐CGGTCCTCCTCAAACTTATC ‐3′
*Atgl*	5′‐GAACCGAAAGACCTGATGAC‐3′	5′‐GAAATTGGGTGACCATCTACC‐3′
*Acox*	5′‐AAGCTTCGTGCAGCCAGATTGGTA‐3′	5′‐AAGGCATCCACCAGAGCAACA‐3′
*Cd36*	5′‐ACGACTGCAGGTCAACATACTGGT‐3′	5′‐TGGTCCCAGTCTCATTTAGCCACA‐3′
*Cpt1*	5′‐GGCTCAAGCTGTTCAAGATA‐3′	5′‐CTCCATGGCTCAGACAATAC‐3′
*Dgat1*	5′‐ACTGGTGGAATGCTGAGTCTGTCA‐3′	5′‐ACAGCTGCATTGCCATAGTTCCCT‐3′
*Dgat2*	5′‐CAAGAAGTTCCCTGGCATAA‐3′	5′‐GTATACCTCATTCTCTCCAAAGG‐3′
*Hsl*	5′‐CACAAGCACTACTGGGATAC‐3′	5′‐GTATCTTCTTCCGTGCCAG‐3′
*Lxrα*	5′‐ATCGTGTCCGTGCAGGAGATTGTT‐3′	5′‐TTAATGAACTCCACCTGCAGCCCT‐3′
*Mtp*	5′‐TCAGGTGCTGGGTGTCACTTCAAA‐3′	5′‐ATTACTCCTGCCACTTGCTTCCCA‐3′
*Mgat2*	5′‐GAGGTTCCGCATCTACAAAC‐3′	5′‐GCCGTCTTTATCGACATTCC‐’3
*Mcad*	5′‐GGCCTTTGCTGGAGATATT‐3′	5′‐CGTAGTTACATGAGGGTGAAG‐3′
*Plin2*	5′‐GAGTCCCTGTCTACCAAGAT‐3′	5′‐CAGAGAGCTTGTCCTGAATTT‐3′
*Plin3*	5′‐GTGGGACAGATGGTGATTAG ‐3′	5′‐CACTCTGCCTGACATTACAC‐3′
*Pgc1α*	5′‐AGTACAACAATGAGCCCGCGAACA‐3′	5′‐TGGCAGGGTTTGTTCTGATCCTGT‐3′
*Pparα*	5′‐AGATCGGCCTGGCCTTCTAAACAT‐3′	5′‐TGTGCAAATCCCTGCTCTCCTGTA‐3′
*Srebp1c*	5′‐GGAGCCATGGATTGCACATTTG‐3′	5′‐GCTTCCAGAGAGGAGCCCAG‐3′
*Tfam*	5′‐GCTGATGGGCTTAGAGAAGGAAG‐3′	5′‐TGCTGACCGAGGTCTTTTTGG‐3′
*Ucp2*	5′‐AAGCAGTTCTACACCAAGGGCTCA‐3′	5′‐AATGGCATTTCGGGCAACATTGGG‐3′

### Statistics

One‐way analysis of variance (ANOVA) was used to test for differences among groups in body weight, food intake, percent body fat, fat pad weight, blood parameters, and mRNA levels of genes examined in this study. Statistical analyses of data were performed in SAS (SAS Institute, Inc., Cary, NC). A significant main effect (*P* < 0.05) was followed‐up with Tukey multiple group comparison. Values are reported as means ± standard error of the mean (SEM), and a *P* value < 0.05 denotes a statistically significant difference.

## Results

### EndEx decreases body weight and improves body composition in OLETF rats

**Figure 1.**
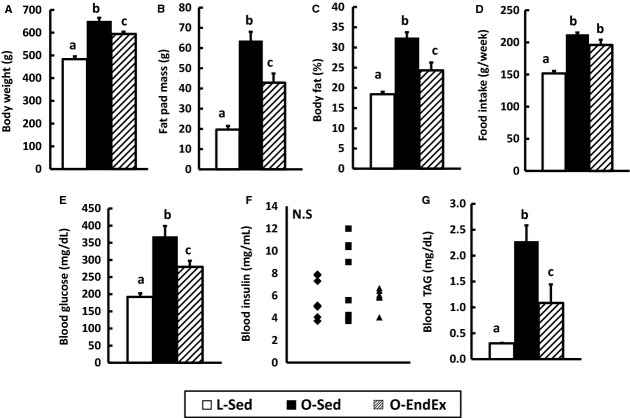
Endurance exercise improves metabolic outcomes in hyperphagic, obese rats. Body weight (A), fat pad mass (B, sum of omental, retroperitoneal, and epididymal pad), and % of body fat (C) were determined at the end of the study. Weekly food intakes (D) were averaged across the period of the intervention (age 20–32 weeks). Levels of fasting blood glucose (E), insulin (F) and triacylglycerol, TAG (G) were determined at the end of the study. Values are means ± SEM (*n* = 6–8 rats/group). In (F), ♦ = L‐Sed rats; ■ = O‐Sed rats; ▲ = O‐EndEx rats. Bars with different letters are significant (*P* < 0.05).

Consistent with our previous reports (Rector et al. 2008, 2011; Linden et al. 2014), O‐Sed rats developed obesity due to hyperphagia compared with L‐Sed rats and O‐EndEx rats had lower body weight (*P* < 0.05; Fig. 1A) and favored body composition (*P* < 0.05; Fig. 1B and C) compared with O‐Sed rats. O‐Sed and O‐EndEx had significantly greater weekly food consumptions than L‐Sed rats (*P* < 0.05; Fig. 1D), with O‐Sed and O‐EndEx rats having similar calorie intake from 20 to 32 weeks of age (Fig. 1D).

### EndEx improves serum parameters in OLETF rats

EndEx training improved fasting glucose compared with O‐Sed (*P* < 0.05; Fig. 1E); however, fasting glucose levels did not return to those observed in L‐Sed. Similarly, EndEx partially attenuated serum TAG compared with O‐Sed rats (*P* < 0.05; Fig. 1G). There was no significant difference in the levels of fasting plasma insulin among the groups (Fig. 1F), likely due to the variability in pancreatic *β*‐cell dysfunction and disease progression observed in the O‐Sed rats.

### EndEx increases mRNA levels for markers of enterocyte lipid anabolism

**Figure 2.**
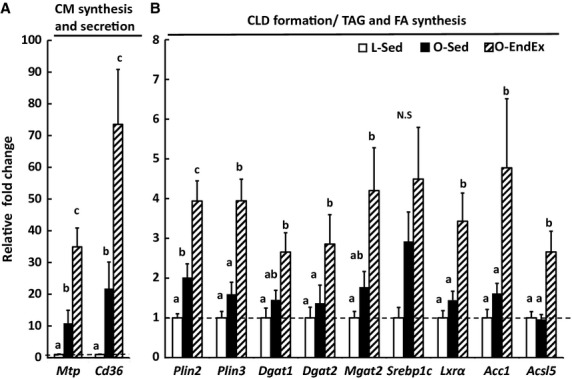
Endurance exercise increases mRNA levels of intestinal genes involved in lipid anabolism in hyperphagic, obese rats. Mucosa from jejunum segment was collected after 12 h fast from indicated rat group at the end of the study. Values are means ± SEM (*n* = 4–8 rats/group). *Mtp*, microsomal triglyceride transfer protein; *Cd36*, fatty acid translocase; *Plin2*, perilipin 2; *Plin3*, perilipin 3; *Dgat1*, diacylglycerol‐O‐acyltransferase 1; *Dgat2*, diacylglycerol‐O‐acyltransferase 2; *Mgat2*, monoacylglycerol‐O‐acyltransferase 2; *Srebp1c*, sterol regulatory element‐binding protein 1; *Lxrα*, liver X receptor *α*;* Acc1*, acetyl‐CoA carboxylase 1; *Acsl5*, acyl‐CoA synthetase 5. Bars with different letters are significant (*P* < 0.05).

Relative mRNA levels for genes associated with lipid anabolism including CM synthesis and secretion (*Mtp* and *Cd36*), CLD formation (*Plin2* and *Plin3*), and fatty acid and TAG synthesis (*Dgat1, Dgat2, Mgat2, Srebp1c, Lxrα, Acc1, and Acsl5*) were determined in intestinal mucosa. O‐Sed rats had higher mRNA levels of *Mtp* and *Cd36* compared with L‐Sed rats (*P* < 0.05; Fig. 2A), which were further increased in O‐EndEx rats (*P* < 0.05; Fig. 2A). While O‐Sed rats had higher mRNA level of *Plin2*, a gene involved in CLD formation, than L‐Sed rats, O‐EndEx rats had even greater mRNA levels of *Plin2* and *Plin3* compared with L‐Sed rats and O‐Sed rats (*P* < 0.05; Fig. 2B). O‐Sed rats had similar mRNA levels for genes in TAG and fatty acid synthesis compared with L‐Sed rats; however, O‐EndEx rats had greater mRNA levels in TAG and fatty acid synthesis genes, including *Dgat2, Lxrα, Acc1, and Acsl5* compared with L‐Sed and O‐Sed rats (*P* < 0.05; Fig. 2B). In addition, O‐EndEx rats had higher mRNA levels of *Dgat1* and *Mgat2* compared with L‐Sed rats (*P* < 0.05; Fig. 2B). There was no significant difference in mRNA level of *Srebp1c* among the groups (Fig. 2B).

### EndEx increases mRNA levels for markers of enterocyte lipid catabolism and mitochondrial content

**Figure 3.**
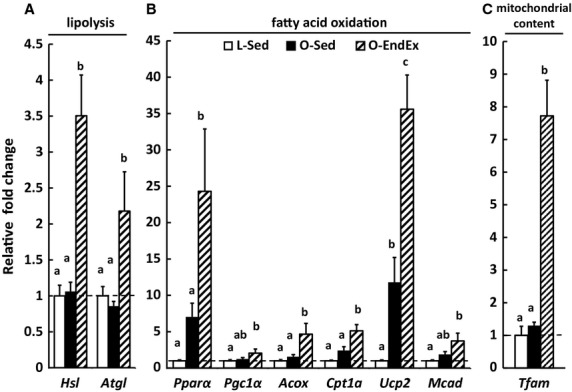
Endurance exercise increases mRNA levels of intestinal genes involved in lipid catabolism and mitochondrial content/function/biosynthesis in hyperphagic, obese rats. Mucosa from jejunum segment was collected after 12 h fasting from indicated rat group at the end of the study. Values are means ± SEM (*n* = 4–8 rats/group). *Hsl*, hormone‐sensitive lipase; *Atgl*, adipose triglyceride lipase; *Pparα*, peroxisome proliferator‐activated receptor *α*;* Pgc1α*, PPAR‐*γ* coactivator‐1 *α*;* Acox*, acyl‐CoA oxidase; *Cpt1*, carnitine palmitoyltransferase I; *Ucp2*, uncoupling protein 2; *Mcad*, medium‐chain acyl‐coenzyme A dehydrogenase; *Tfam*, mitochondria transcription factor A. Bars with different letters are significant (*P* < 0.05).

Gene expression of markers related to catabolic metabolism of TAG and fatty acids including lipolysis (*Hsl* and *Atgl*) and FAO (*Pparα, Pgc1α, Acox, Cpt1α, Ucp2,* and *Mcad*) and mitochondrial content (*Tfam*) were determined in intestinal mucosa. O‐Sed and L‐Sed rats had similar mRNA levels of *Hsl* and *Atgl* (Fig. 3A); whereas, EndEx increased these lipolytic genes (*P* < 0.05; Fig. 3A). O‐Sed rats had higher mRNA level of *Ucp2* compared with L‐Sed rats; however, O‐EndEx rats had higher mRNA levels in genes related to FAO including *Pparα, Acox, Cpt1,* and *Ucp2* compared with L‐Sed and O‐Sed rats (*P* < 0.05; Fig. 3B). O‐EndEx rats also had higher mRNA levels of *Pgc1α* and *Mcad* compared with L‐Sed rats only (*P* < 0.05; Fig. 3B), and EndEx significantly increased mRNA levels of *Tfam*, a marker of mitochondrial content, compared with L‐Sed and O‐Sed (*P* < 0.05; Fig. 3C).

## Discussion

Here, we demonstrate that EndEx training significantly alters mRNA levels for genes involved intestinal lipid metabolism. While HIO increased intestinal mRNA levels of lipid metabolism genes, including *Mtp*,* Cd36*,* Plin2,* and *Ucp2,* in O‐Sed compared to L‐Sed rats, EndEx training robustly increased mRNA levels of even more genes involved in intestinal lipid anabolism and catabolism as well as mitochondrial content/function/biogenesis, including *Plin*s, *Dgat2*,* Acc1*,* Pparα*,* Cpt1*,* Acox,* and *Tfam*. These findings suggest potential for enhanced lipid turnover and more efficient FAO for energy utilization within enterocytes with EndEx. Overall, this study provides evidence that EndEx programs intestinal lipid metabolism, likely contributing to its role in improving metabolic outcomes in obesity and type 2 diabetes.

EndEx was previously found to reverse elevated lymphatic lipid transport found in OLETF rats (Hayashi et al. 2002). Because of this, we hypothesized that changes in the intestine in response to EndEx may limit substrates for TAG synthesis and secretion on CMs. In fact, previous studies found that EndEx increases FAO in tissues including muscle, liver, and adipose (Laye et al. 2009; Rector et al. 2010b, 2011), and this increase in FAO activity is accompanied by higher mRNA levels of genes associated with FAO and/or increased mitochondrial content and function. Consistent with these findings, the intestine of O‐EndEx rats had remarkably higher mRNA levels of *Pparα* and its downstream targets *Acox*,* Cpt1,* and *Ucp2*, compared to O‐Sed rats. Although activity of intestinal FAO is considered low, PPAR*α* is highly expressed in the small intestine (Bunger et al. 2007; de Vogel‐van den Bosch et al. 2008) and PPAR*α*‐mediated FAO in enterocytes has previously been shown to reduce the postprandial triglyceridemic response by limiting substrates for TAG synthesis and secretion in CMs (Kimura et al. 2011, 2013; Uchida et al. 2011). Additionally, O‐EndEx rats had remarkably higher mRNA level of *Tfam,* which was reported to directly correlate with mitochondrial DNA content (Ekstrand et al. 2004). Unexpectedly, O‐EndEx rats had increased mRNA levels of *Cd36* and *Mtp,* genes typically associated with CM synthesis and secretion, compared with O‐Sed and L‐Sed rats. It remains unclear why these genes may be elevated; however, these genes may also play roles in other metabolic pathways within enterocytes independent of CM synthesis and secretion. Taken together, our finding suggests that EndEx may increase FAO in the small intestine and contribute to the previously reported reduced postprandial triglyceridemic response (Hayashi et al. 2002). Future studies are necessary to determine whether the changes observed in mRNA levels in these processes translate to physiological changes in FAO in the small intestine.

Surprisingly, while O‐EndEx rats had higher intestinal mRNA levels of genes involved intestinal catabolism (lipolysis and FAO), they also had higher intestinal mRNA levels of genes involved in intestinal anabolism (CLD formation, TAG synthesis, and fatty acid synthesis) compared with O‐Sed rats. These findings differ from those previously observed in the liver and adipose tissue of these animals (Rector et al. 2008, 2010b; Laye et al. 2009); however, similar findings have been observed in skeletal muscle with exercise training (Goodpaster et al. 2001; Koves et al. 2013). In skeletal muscle, parallel activation in lipid anabolism and catabolism suggests that intramuscular CLDs are able to provide a rapid, local energy source in response to exercise. Based on our findings related to intestinal lipid metabolism and mitochondrial content, EndEx may provide similar adaptations by programming intestinal lipid metabolism to be more efficient under certain conditions. In fact, the small intestine accounts for 25% of total body oxygen consumption (Yen et al. 1989; Vaugelade et al. 1994), reflecting the substantial energy demand by the intestine for nutrient processing and other basic cellular functions. Although glutamine is considered the major fuel source for enterocytes (Windmueller and Spaeth 1980), it has also been demonstrated that enterocytes oxidize fatty acids during lipid absorption (Gangl and Ockner 1975). It is possible that more efficient lipid metabolism within enterocytes may provide an efficient/extra energy source to process overloaded nutrients and limit TAG secretion from the intestine in obesity and type 2 diabetes, contributing to the hypotriglyceridemic effect of EndEx seen in other studies (Merrill et al. 1989; Hardman 1998; Davitt et al. 2013). In addition, the efficient/extra energy source produced from lipid metabolism within enterocytes may be required for EndEx to carry out other beneficial functions on small intestine to improve obesity and diabetes such as enhancing the release of glucagon‐like peptide 1 (GLP‐1; Allen et al. 2012) and cholesterol efflux (Khabazian et al. 2009).

The mechanism for how EndEx may alter intestinal lipid metabolism is not clear and needs to be identified in future studies. The EndEx‐induced changes in intestinal lipid metabolism could be secondary or due to crosstalk between tissues that are modified by EndEx. For example, EndEx was reported to alter metabolism of thyroid hormone and leptin in rats (Story and Griffith 1974; Uribe et al. 2014). Both thyroid hormone and leptin have been demonstrated to have effects on lipid metabolism in other tissues and have receptors and signaling present in the small intestine. In addition, IL‐6, a factor released from exercise trained muscle, enhances the release of GLP‐1 by the intestine which contributes to improved glycemia under obesity and diabetes (Pedersen et al. 2004; Allen et al. 2012). Recently, emerging evidence also shows that exercise training can modify the composition of gut microbiota and have beneficial effects to improve obesity (Evans et al. 2014; Petriz et al. 2014). Each of these examples may serve as potential mechanisms of EndEx‐induced changes in intestinal lipid metabolism.

The effect of obesity and type 2 diabetes on intestinal lipid metabolism has been investigated in a variety of models including, high fat feeding, high fat/fructose/cholesterol feeding, and genetic models, such as leptin‐deficiency (Naples et al. 2012; Uchida et al. 2012), however, little is known about the effects of hyperphagia‐induced obesity and type 2 diabetes on intestinal lipid metabolism. Here, HIO increased intestinal mRNA levels of genes involved in CM synthesis and secretion (*Cd36* and *Mtp)* but not in genes involved in TAG and fatty acid synthesis. Higher levels of *Cd36* and *Mtp* are consistent with greater lymphatic lipid secretion previously reported in the OLETF rat model of HIO (Hayashi et al. 2002). Multiple genes involved in TAG synthesis (*Mgat2* and *Dgat2*) and FAO (*Acox*,* Cpt1*,* Pparα*) have been shown to be elevated in high‐fat‐diet fed, obese, compared with low fat fed, lean mice (de Wit et al. 2008; Uchida et al. 2012), whereas, in the current report O‐Sed rats fed standard chow only had higher level of one gene in FAO, *Ucp2,* in enterocytes compared with L‐Sed rats. This suggests that intestinal genes involved in TAG synthesis and FAO may be less sensitive to the metabolic changes in obesity than to changes in diet composition. In addition, the pool of TAG stored in CLD is a possible source of lipid contributing to CM synthesis and secretion. In this study, O‐Sed rats had higher intestinal *Plin2*, a CLD associated protein associated with large CLDs in enterocytes (Lee et al. 2009) and cellular TAG content, compared with L‐SED rats.

Overall, our study provides novel evidence that HIO and EndEx program intestinal lipid metabolism in OLETF rats. This is the first study demonstrating that EndEx training increases mRNA expression of genes involved in intestinal lipid anabolism and catabolism as well as mitochondrial biogenesis/content within enterocytes. We propose that the exercise‐induced changes in lipid metabolism and mitochondria biogenesis allow more efficient FAO in enterocytes and this altered lipid metabolism may contribute to the therapeutic effects of exercise training on treating obesity and type 2 diabetes. In addition, we found that the magnitude of changes in mRNA expression in enterocytes is much larger than previously identified changes in other tissues involved in lipid metabolism in response to EndEx (Rector et al. 2010b; Yasari et al. 2010), highlighting the potential importance of exercise‐induced alterations in intestinal lipid metabolism. Future studies are required to know if the changes in gene expression involved in intestinal lipid metabolism reflect significantly physiological performance and to investigate the molecular mechanism of EndEx regulating intestinal lipid metabolism.

## Acknowledgments

The authors gratefully acknowledge the excellent technical assistance of P. Thorne and G. Meers and M.H. Laughlin for partial financial support of animal husbandry. We also thank E. Gibson, B. Muller, K. Tacchi, M. Brielmaier, and N. Fleming for all of their hard work as animal trainers.

## Conflicts of Interest

None declared.
